# GnRH regulates the expression of its receptor accessory protein SET in pituitary gonadotropes

**DOI:** 10.1371/journal.pone.0201494

**Published:** 2018-07-27

**Authors:** Charlotte Avet, Chantal Denoyelle, David L’Hôte, Florence Petit, Céline J. Guigon, Joëlle Cohen-Tannoudji, Violaine Simon

**Affiliations:** Sorbonne Paris Cité, Université Paris-Diderot, CNRS UMR 8251, INSERM U1133, Biologie Fonctionnelle et Adaptative, Physiologie de l’axe gonadotrope, Paris, France; Universite de Rouen, FRANCE

## Abstract

Reproductive function is under the control of the neurohormone GnRH, which activates a G-protein-coupled receptor (GnRHR) expressed in pituitary gonadotrope cells. GnRHR activates a complex signaling network to regulate synthesis and secretion of the two gonadotropin hormones, luteinizing hormone and follicle-stimulating hormone, both regulating gametogenesis and steroidogenesis in gonads. Recently, in an attempt to identify the mechanisms underlying GnRHR signaling plasticity, we identified the first interacting partner of GnRHR, the proto-oncogene SET. We showed that SET binds to intracellular domains of GnRHR to enhance its coupling to cAMP pathway in αT3-1 gonadotrope cells. Here, we demonstrate that SET protein is rapidly regulated by GnRH, which increases SET phosphorylation state and decreases dose-dependently SET protein level. Our results highlight a post-translational regulation of SET protein involving the proteasome pathway. We determined that SET phosphorylation upon GnRH stimulation is mediated by PKC and that PKC mediates GnRH-induced SET down-regulation. Phosphorylation on serine 9 targets SET for degradation into the proteasome. Furthermore, a non-phosphorylatable SET mutant on serine 9 is resistant to GnRH-induced down-regulation. Altogether, these data suggest that GnRH-induced SET phosphorylation on serine 9 mediates SET protein down-regulation through the proteasome pathway. Noteworthy, SET down-regulation was also observed in response to pulsatile GnRH stimulation in LβT2 gonadotrope cells as well as *in vivo* in prepubertal female mice supporting its physiological relevance. In conclusion, this study highlights a regulation of SET protein by the neurohormone GnRH and identifies some of the mechanisms involved.

## Introduction

Reproductive function is under the control of the hypothalamic neurohormone gonadotropin-releasing hormone (GnRH), which activates an heptahelical transmembrane receptor expressed at the surface of pituitary gonadotrope cells, the GnRH receptor (GnRHR). Activation of GnRHR leads to synthesis and secretion of the two gonadotropin hormones, luteinizing hormone (LH) and follicle-stimulating hormone (FSH), both regulating gametogenesis and steroidogenesis in gonads. GnRHR activates a complex signaling network involving notably calcium and cyclic AMP (cAMP) pathways. GnRHR is mainly coupled to phospholipase Cβ *via* Gαq/11, leading to a rapid increase of intracellular calcium concentrations and activation of several PKC isoforms [1]. PKC regulates gene transcription directly or through activation of the MAPK cascade. Recruitment of the cAMP/PKA pathway by GnRH also contributes to the regulation of a limited set of gene expression including *Gnrhr* [2,3].

G-protein-coupled receptors (GPCR) interact with GPCR interacting proteins (GIP), which influence signal transfer from receptor to G proteins, receptor trafficking between plasma membrane and intracellular compartments or subcellular localization [4,5]. Recently, a novel GIP, the proto-oncogene SET, was discovered. This was initially discovered by showing that SET, through its interaction with the third intracellular domain of M3 muscarinic receptor, inhibits signal transfer from the receptor to Gαq/11 protein [6,7]. Later on, we and others identified three other GPCR regulated by SET, the β1-adrenergic receptor [8], the type 1A angiotensin receptor [9] and the GnRHR [3]. We showed that SET binds to intracellular domains of GnRHR and induces a signaling switch from calcium to cAMP pathways, which increases the transcriptional activity of *Gnrhr* [3]. SET was initially described as part of the *Set-can* fusion gene, a putative oncogene associated with acute undifferentiated leukaemia [10]. SET regulates gene transcription through multiple mechanisms including chromatin remodeling [11], histone chaperone activity [11,12], epigenetic modifications of histones [13,14] or association with factors of the transcriptional machinery including histone acetyltransferases [15,16], transcription factors and nuclear receptors [17,18] In addition, SET, also called I2PP2A (inhibitor 2 of protein phosphatase 2A), regulates many cellular processes such as cell cycle and proliferation [19,20,21], segregation of sister chromatid during meiosis [22,23], apoptosis [24,25] and cell migration [26] through inhibition of PP2A activity [27].

SET is ubiquitously expressed in mammals [10,28] and we previously detected it in pituitary gonadotrope cells [3]. Although SET plays a critical role in many cellular processes, mechanisms regulating its expression and activity are poorly known. Several studies in the brain [29,30], gonads [31] and kidney [32] have reported that SET is highly expressed during embryogenesis and that its expression decreases at birth. Deregulations of SET expression during adulthood have only been described in some pathological conditions such as cancers and Alzheimer’s disease and their origins are still unknown [32,33,34,35].

Here we demonstrate, by using both *in vitro* approaches with gonadotrope cell lines and an *in vivo* mouse model, that the neurohormone GnRH is responsible for SET protein down-regulation in gonadotrope cells through mechanisms occurring at the post-translational level.

## Materials and methods

### Materials and antibodies

GnRH agonist ([D-Trp^6^]GnRH, triptorelin), poly-L-lysine, GF109203X and proteasome inhibitor MG132 were purchased from Sigma-Aldrich Chemie (Saint-Quentin Fallavier, France). The proteasome inhibitor clasto-lactacystin–Lactone was purchased from Merck Millipore. Protease inhibitors (Complete Mini, EDTA-free) and phosphatase inhibitors were from Roche Diagnostics (France). DMEM was from Lonza (Verviers, Belgium). Superscript II reverse transcriptase and proteinase K were from Invitrogen (Cergy Pontoise, France). Foetal bovine serum was from Pan Biotech GmbH (Dutscher, Les Ulis, France). The QuickChange site-directed mutagenesis kit was purchased from Agilent technologies (Massy, France). The phosphoprotein Enrichment Kit was obtained from Qiagen (Courtaboeuf, France). For antibodies, the rabbit polyclonal anti-SET antibody was kindly provided by Dr TD Copeland (National Cancer Institute-Frederick, MA), mouse monoclonal anti-vinculin (#hVIN-1, catalog no. V9131, Research Resource Identifier (RRID: AB_477629) was from Sigma-Aldrich, rabbit polyclonal anti-total ERK1/2 (catalog no. 9102, RRID: AB_330744) was from Cell Signaling and rabbit anti-GAPDH (catalog no. sc-25778, RRID: AB_10167668) was from Santa Cruz.

### Cell culture

Pituitary gonadotrope cell lines αT3-1 and LβT2 generated by Pamela Mellon (LaJolla, CA, USA; [36]) were grown in DMEM containing 10% of foetal bovine serum (FBS) and 0.5% penicillin/streptomycin (PS). Cells were passaged weekly and incubated at 37°C in a humidified atmosphere with 5% CO_2_.

### Animals and treatments

Studies were conducted on C57BL/6JRj mice aged 7 to 17 days postnatal born at the animal facility from genitors purchased at Janvier Labs (Le Genest St Isle, France). Mice were maintained under controlled conditions (12h light/12h dark cycle) with food (Scientific Animal Food and Engineering (SAFE), A03-10) and water available *ad libitum*. The day of birth was designed as 0 dpn. Mice were anesthetized with a mix of ketamine (Imalgene® 1000) and xylazine (Rompun® 2%) and then killed by cervical dislocation. Pituitaries were collected, frozen in liquid nitrogen and stored at -80°C for RNA and protein extraction. Ten μg of Ganirelix acetate (Orgalutran®, N.V. Organon, Puteaux, France) or saline was subcutaneously injected twice on prepubertal female mice at 12 and 13 dpn, following a procedure previously published [37]. Experiments were performed in accordance with standard ethics guidelines and were approved by Institutional Animal care and Use committee of the University Paris Diderot and by the French Ministry of Agriculture (agreement #04015.01).

### SET protein expression

Gonadotrope cells were cultured in 12-well plates (10^6^ cells/well) in DMEM containing 10% FBS and 0.5% PS. Cells were starved overnight in serum-free DMEM and incubated for the indicated times with increasing concentrations of the GnRH agonist, triptorelin (GnRHa). Gonadotrope cells were washed after GnRHa treatment with ice-cold PBS and homogenized in a buffer containing 10 mM Tris-HCl (pH 7.4), 30 mM sodium pyrophosphate, 50 mM NaCl, 1% TritonX-100, and 1 mM dithiothreitol (DTT) supplemented with protease and phosphatase inhibitors cocktails. Homogenates were centrifuged at 20,000 × g for 30 min at 4°C. Protein extracts (20 μg) were separated on a 10% SDS-PAGE. After electrotransfer onto nitrocellulose membrane, SET was immunodetected using polyclonal anti-SET antibody (dilution 1:2,000) and enhanced chemiluminescence detection system (ECL+; GE Healthcare). Blots were analyzed with a Fuji LAS-4000 imager and quantified with MultiGauge software analysis. Vinculin was used as internal loading control for SET expression (dilution 1:20,000). For evaluation of proteasome implication, gonadotrope cells were pre-treated with the vehicle control DMSO or the proteasome inhibitors MG132 (2 hours, 3 μM) or clasto-lactacystin–Lactone (2 hours, 10 μM) before GnRHa stimulation.

Pooled (7 dpn) or single (12–17 dpn) frozen pituitaries were lysed with TissueLyser (Qiagen) in lysis buffer (10 mM Tris HCl, 50 mM NaCl, 1% Triton, 30 mM NaPPi, 50 mM NaF, 5μM ZnCl_2_, 100μM Na_3_VO_4_, 1 mM DTT, 1X protease inhibitor (Roche), 1X phosphatase inhibitor [Thermo Scientific]) following a protocol described elsewhere [37]. Twenty μg of lysates were used for the determination of SET and GAPDH (dilution 1:3,000) used as a loading control. Proteins were detected by chemiluminescence with ECL, analyzed with a Fuji LAS-4000 imager and quantified with MultiGauge software analysis.

### mRNA quantification by real-time RT-PCR

Total RNA from gonadotrope cells was isolated with a RNeasy-kit (QIAGEN). First-strand cDNA was obtained from 2 μg RNA with Superscript II reverse transcriptase according to the manufacturer's instructions. For *Set*, *Lhb* and *Fshb* mRNA level quantification, real-time polymerase chain reaction (PCR) was carried out in the LightCycler 480 Instrument (Roche Diagnostics, Meylan, France) using 4.5 μl of Takyon No ROX SYBR master mix (Eurogentec), 5 μl of a 1:10 to 1:160 cDNA dilutions, and 0.5 μM of primer (*Set* forward primer, 5’-GTGACCCGTCTTCAAAGTCC-3’; *Set* reverse primer, 5’-AAGCTCTCTGGCTCTTCGTG-3’; *Lhb* forward primer, 5’-ATCACCTTCACCACCAGCAT-3’; *Lhb* reverse primer, 5’-GACCCCCACAGTCAGAGCTA-3’; *Fshb* forward primer, 5’-GTGCGGGCTACTGCTACACT-3’; *Fshb* reverse primer, 5’-CAGGCAATCTTACGGTCTCG-3’). PCR product migrated as a single band on ethidium bromide gel. Expression levels were normalized to *Hprt* mRNA used as an internal control (forward primer, 5’-AGGACCTCTCGAAGTGT-3’; reverse primer, 5’-ATTCAAATCCCTGAAGTACTCAT-3’). Data were analyzed using the advanced-E-method with standard curve-derived efficiencies obtained from LightCycler 480 software (Roche).

Pooled (7 dpn) or single (12–17 dpn) frozen pituitaries were lysed with TissueLyser (Qiagen) and RNA was obtained using RNeasy mini kit (Qiagen), following manufacturer’s protocol, as previously described [37] Total RNA (100 ng) was reverse transcribed with Superscript II (Invitrogen) and random primers (Invitrogen) following manufacturer’s instructions. Relative quantification of SET mRNA was performed following normalization to *Hprt* used as a reference gene. Real-time PCR was performed with Lightcycler® 480 SYBR Green I Master (Roche Molecular Biochemicals, La Rochelle, France) and Light Cycler instrument (Roche).

### SET phosphorylation

For phosphorylation experiments, αT3-1 gonadotrope cells were cultured in 6-well plate (8 x 10^6^ cells/2 ml/well) in complete medium. Cells were starved overnight in serum-free medium and the next day, confluent cells were washed three times in phosphate-free Krebs/HEPES buffer (10 mM HEPES; 118 mM NaCl; 4.3 mM KCl; 1.17 mM MgSO_4·_ 7H_2_O; 1.3 mM CaCl_2_; 25 mM NaHCO_3_ and 11.7 mM glucose, pH 7.4) and incubated in phosphate-free Krebs/HEPES buffer supplemented with [^32^P] orthophosphate (50 μCi/ml) for 1 hour at 37°C. Cells were stimulated with the GnRHa (100 nM) for 0.5 hour, and reactions were terminated by rapid aspiration of the drug-containing medium and application of 1 ml of phosphate-free Krebs/HEPES buffer. Cells were then collected and centrifuged at 500 x g (5 min) and lysed in 100 μl of ice-cold lysis buffer (50 mM Tris-HCl; 10 mM EDTA; 100 mM NaCl; 0.5% Triton-X100; 1 mM DTT; phosphatase and protease inhibitor mixtures, pH 7.4). Cell lysates were incubated 1 hour at 4°C and centrifuged at 12,000 x g (15 min, 4°C). Supernatants were pre-cleared with protein A-Sepharose beads and then incubated (~300 μg of protein in 100 μl of lysis buffer) with polyclonal anti-SET antibody (dilution 1:100) for 16 h at 4°C. Protein A-Sepharose beads (40 μl) were added, and incubation was continued for 2 hours. The resin was pelleted and washed three times with lysis buffer. Immunoprecipitated proteins were eluted with 2X loading buffer (120 mM Tris-HCl, pH 6.8; 20% glycerol; 4% SDS; 20% β-mercaptoethanol), subjected to SDS-PAGE and transferred to a nitrocellulose membrane. Phosphorylated SET was visualized and quantified by autoradiography using a Fuji Phosphoimager FLA7000. Levels of total SET protein were examined by immunoblotting with anti-SET antibody (dilution 1:2,000) to normalize the level of phosphorylated SET.

Phosphoprotein enrichment was carried out using Qiagen columns according to the manufacturer’s instructions. Briefly, gonadotrope cells (2 x 10^7^ cells) were incubated or not with GnRHa (100 nM, 0.5 hour) and lysed with a PhosphoProtein lysis buffer containing 0.25% CHAPS, protease inhibitor cocktail and 50 U/ml of Benzonase®Nuclease. The lysates were incubated 30 min at 4°C and centrifuged at 12,000 x g at 4°C for 40 min. Protein from supernatant (1.1 mg) were adjusted at a 0.1 mg/ml concentration using the PhosphoProtein lysis buffer containing 0.25% CHAPS and were then loaded onto the PhosphoProtein Purification Column at RT. After washing with the PhosphoProtein lysis buffer (6 ml), the bound phosphoproteins were eluted with 2.5 ml of elution buffer. Proteins eluted were concentrated with Nanosep ultra filtration column and centrifugation at 10,000 x g for 10 min. Equal amount of phosphoprotein extracts were loaded on 10% SDS-PAGE, transferred on nitrocellulose membrane and immunoblotted with anti-SET antibody. Phosphorylated ERK was detected using anti-total ERK1/2 antibody (dilution 1:1,000) to monitor activation of GnRHR signaling. When indicated, cells were pre-incubated with 2 μM of the PKC inhibitor, GF109203X, 1 hour prior to GnRHa stimulation and phosphoproteins purification.

### Expression of SET mutants on serine 9 in gonadotrope cells

SET mutants on serine 9 were obtained from pCDNA3-His-SET WT by site-directed mutagenesis. αT3-1 gonadotrope cells (3 x 10^6^ cells/tip) were electroporated with 10 μg of pCDNA3.1-His-SET WT, pCDNA3-His-SET A9 or pCDNA3-His-SET E9 using the Neon transfection system (Invitrogen) according to the manufacturer’s protocol at 1500 mV for 30 ms. Electroporated cells were seeded in 6-well plates in complete medium without antibiotics and 5 hours later, medium was replaced with complete medium. Forty-eight hours later, expression of His-SET protein was measured with SET antibody as described in section “SET protein expression”. His-SET proteins migrate on SDS-PAGE at a higher molecular weight than endogenous SET allowing their specific quantification. In the case of the MG132 treatment, cells were pre-treated 30 hours after transfection with the proteasome inhibitor (10 μM, 18 hours) before cell harvesting.

### Perifusion study

The perifusion system was designed according to Bedecarrats and Kaiser [38] with modifications. Briefly, LβT2 cells were plated at 0.5 x 10^6^ cell/cm^2^ in polydimethylsiloxane perifusion chambers mounted on glass slides coated with poly L-Lysine (250 μg/ml). The cells were incubated in the chambers overnight in a static culture system in DMEM containing 10% FBS and 0.5% PS, then serum starved for 24 hours in DMEM with 1% FBS and 0.1% PS. The chambers were then mounted in the perifusion system and perifused with DMEM containing 1% FBS and 0.1% PS at a constant flow rate of 0.3 ml/min. Cells were treated with medium alone or with pulsatile GnRH (10 nM) at varying frequencies (one pulse every 0.5 hour or one pulse every 2 hours). At the end of the incubations, proteins and mRNA were prepared as described in sections “SET protein expression” and “SET mRNA quantification by real-time RT-PCR”. For *Lhb* and *Fshb* mRNA level quantification, real-time polymerase chain reaction (PCR) was carried out in the LightCycler 480 Instrument (Roche Diagnostics, Meylan, France) using 4.5 μl of Takyon No ROX SYBR master mix (Eurogentec), 5 μl of a 1:10 to 1:160 cDNA dilutions, and 0.5 μM of primer (Lhb forward primer, 5’- ATCACCTTCACCACCAGCAT-3’; Lhb reverse primer, 5’- GACCCCCACAGTCAGAGCTA -3’, Fshb forward primer 5’- GACAGCTGACTGCACAGGAC -3’, Fshb reverse primer, 5’- CAATCTTACGGTCTCGTATACC -3’).

### Statistical analyses

Results are expressed as mean ± SEM. Statistical analyses were performed using a Student's t test for unpaired groups for comparison of two conditions and using a one- or two-way analysis of variance (ANOVA) test followed by the appropriate *post hoc* test (Dunnett’s, or Tukey’s *post hoc* analysis) when more than two conditions were compared. Values were considered statistically different when p was <0.05. Statistical analysis was performed using Prism 6 (GraphPad Software Inc.).

## Results

### GnRH down-regulates SET protein expression in αT3-1 gonadotrope cells

**Fig 1:**
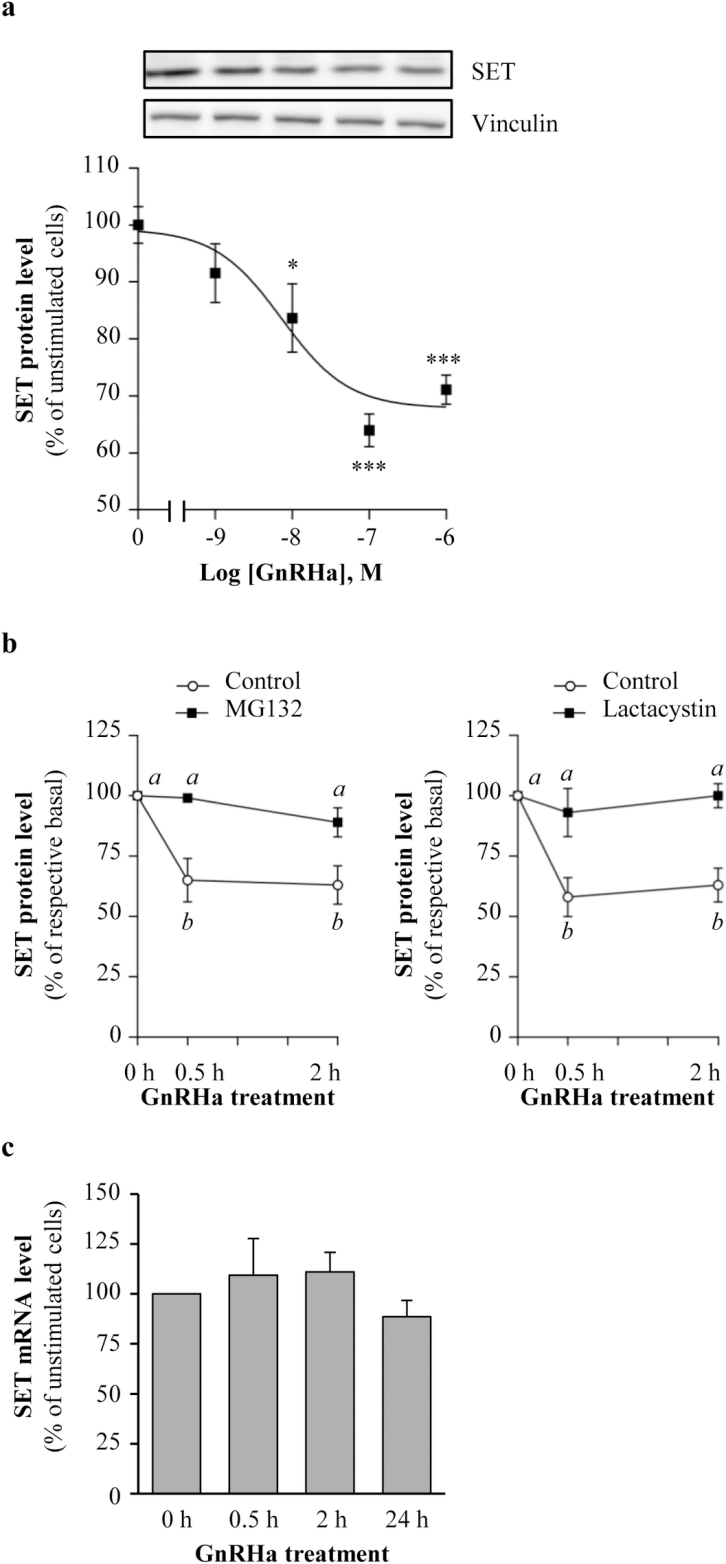
GnRHa down-regulates SET protein expression in αT3-1 gonadotrope. (a) **α**T3-1 gonadotrope cells were incubated during 2 hours with increasing GnRHa concentrations (10^−9^ to 10^−6^ M) and SET protein level was determined by Western blotting. A representative immunoblot of SET expression is shown. Results are normalized by vinculin signals and are expressed as the percentage of SET protein level in absence of GnRHa. Results are expressed as mean ± SEM from 3 to 4 independent experiments. Data were analyzed by One-way ANOVA followed by Dunnett’s test, *: p<0.01 and ***: p<0.001, compared to no GnRHa. (b) **α**T3-1 gonadotrope cells were pre-treated or not (control) with the proteasome inhibitors MG132 (MG132, 2 hours, 3 μM) or the clasto-lactacystin–Lactone (Lactacystin, 2 hours, 10 μM) before stimulation with GnRHa (100 nM) for the indicated times (0.5 and 2 hours). SET protein level was determined by Western blotting and normalized by vinculin signals. SET protein level in GnRHa stimulated cells was expressed as the percentage of respective basal SET expression levels at each time point in the presence or absence of MG132 or clasto-lactacystin–Lactone. Results are expressed as mean ± SEM from 3 to 5 independent experiments. Data were analyzed by Two-way ANOVA followed by Tukey’s test. Distinct letters indicate significant differences between treatments (p<0.05). (c) αT3-1 gonadotrope cells were incubated with GnRHa (100 nM) for increasing periods of time and SET mRNA levels were determined by real-time RT-PCR and normalized to the mRNA levels of *Hprt*. Results are expressed as the percentage of SET mRNA level in unstimulated cells (0 h). Results are expressed as mean ± SEM from 3 independent experiments. Data were analyzed by One-way ANOVA, no significant.

We previously reported that SET interacts with intracellular domains of GnRHR and favors its coupling to the cAMP pathway in the **α**T3-1 gonadotrope cells [3]. In the present study, we asked whether GnRH could modulate SET protein cellular expression. We treated **α**T3-1 gonadotrope cells with increasing concentrations of GnRH agonist, triptorelin (GnRHa, 10^−9^ to 10^−6^ M, 2 hours) and we measured SET protein expression level by Western blotting. We observed that GnRHa dose-dependently decreased SET protein expression with an EC_50_ of 7.3 ± 0.9 nM and a maximal decrease of 36 ± 3% at 10^−7^ M of GnRHa, that was maintained at 10^−6^ M of GnRHa (Fig 1A).

### GnRH induces targeting of SET protein into proteasome

To elucidate the mechanisms involved in SET protein down-regulation upon GnRHa stimulation, we first determined the kinetics of GnRHa action on SET protein expression in αT3-1 cells. We observed that as early as 0.5 hour after GnRHa treatment, SET protein level was significantly reduced by approximately 35% (Fig 1B, 65 ± 9% compared to control cells). This decrease was maintained at 2 and 24 hours of GnRHa stimulation (Fig 1B, 63 ± 8% and 79 ± 7% compared to unstimulated cells, respectively). Changes in SET protein level were not accompanied by any significant changes in SET mRNA level (Fig 1C), indicating that GnRHa-induced SET protein down-regulation does not result from a decrease in SET mRNA transcription and/or stability but rather from a post-transcriptional mechanism. We pre-treated gonadotrope cells with a potent proteasome inhibitor MG132 and determined its impact on GnRHa-induced SET down-regulation. MG132 alone did not significantly affect SET basal expression at any incubation time (91 ± 11% and 87 ± 12% of control cells without MG132 at 0.5 and 2 hours, respectively). In contrast, blocking proteasome with MG132 significantly prevented SET protein down-regulation at 0.5 hour (65 ± 9% *vs*. 99 ± 1% of unstimulated cells without and with MG132, respectively, Fig 1B) and at 2 hours (63 ± 8% *vs*. 89 ± 6% of unstimulated cells without and with MG132, respectively; Fig 1B, *left panel*). The use of another proteasome inhibitor, the clasto-lactacystin–Lactone, also completely blocked GnRHa-induced SET protein down-regulation (Fig 1B, *right panel*). Indeed, in presence of the inhibitor, SET protein down-regulation induced by GnRHa was prevented at 0.5 hour and 2 hours (63 ± 9% *vs*. 93 ± 10% of unstimulated cells without and with lactacystin and 63 ± 7% *vs* 100 ± 5% of unstimulated cells without and with lactacystin, respectively). Altogether, these data show that SET protein is rapidly targeted to the proteasome pathway for degradation following GnRHa stimulation.

### GnRH induces SET phosphorylation in gonadotrope cells

**Fig 2:**
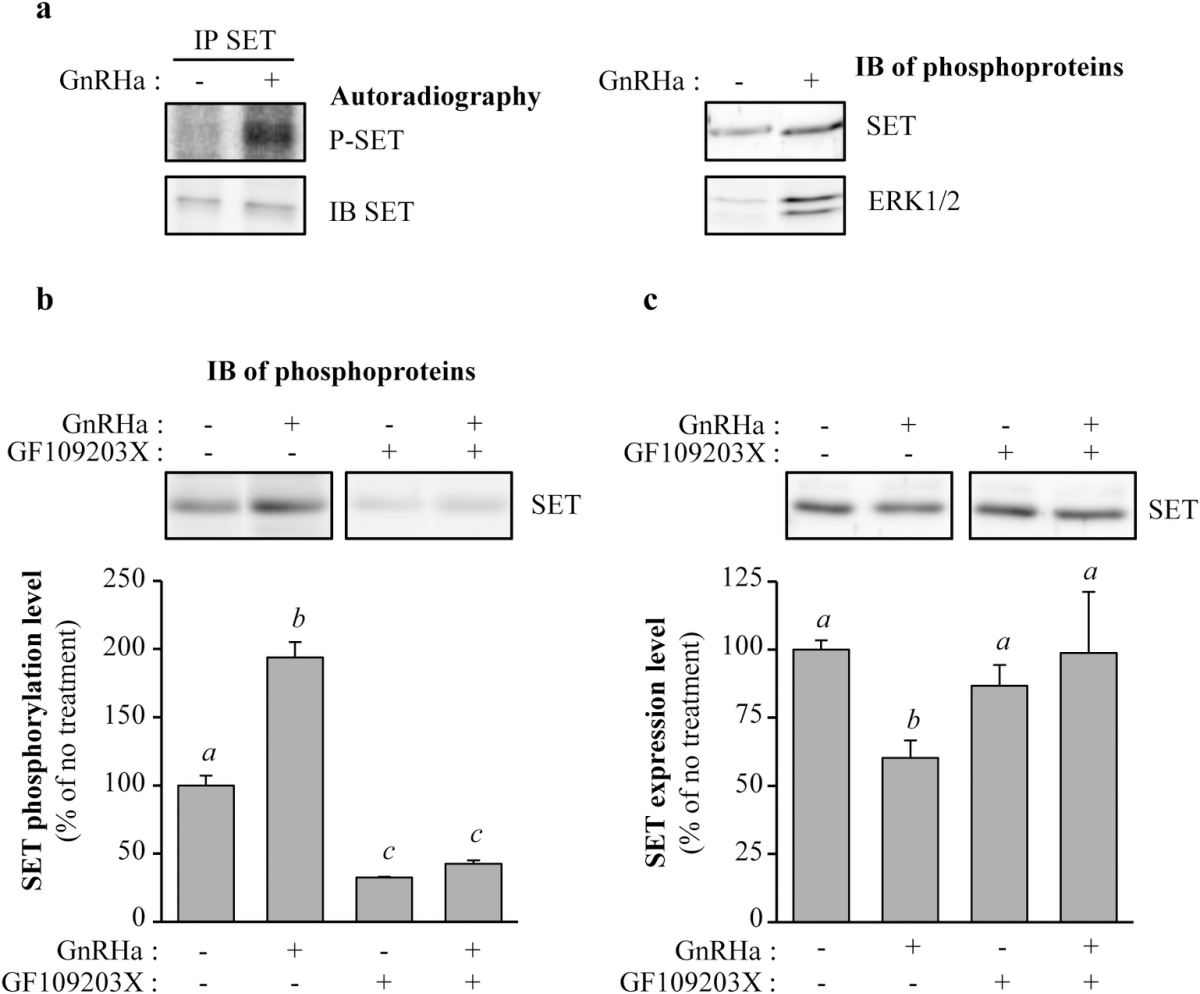
GnRHa increases SET phosphorylation–Involvement of PKC. (a) *Left panel–*αT3-1 gonadotrope cells were plated in 6-well plates and labelled with [^32^P]-orthophosphate (50 μCi/ml) as described in “Materials and methods”. Cells were stimulated (+) or not (-) with GnRHa (100 nM) for 0.5 hour followed by SET immunoprecipitation (IP SET) as described in “Materials and methods”. Immunoprecipitated SET was resolved on 10% SDS-PAGE, electrotransferred and probed with anti-SET antibody (IB SET). Phosphorylated SET (P-SET) was visualized by autoradiography using a Fuji Phosphoimager FLA7000. *Right panel–*αT3-1 gonadotrope cells were incubated (+) or not (-) with GnRHa (100 nM, 0.5 hour) and phosphoproteins were purified by chromatography as described in “Materials and methods”. Phosphorylated SET and ERK1/2 were detected by Western blotting using anti-SET and anti-total ERK1/2 antibodies, respectively. Results are representative of 4 independent experiments. (b) αT3-1 gonadotrope cells were pre-incubated or not with the PKC inhibitor GF109203X (2 μM, 1 hour) prior to GnRHa stimulation (100 nM, 0.5 hour). Phosphoproteins were purified by chromatography as described in “Materials and methods” and phosphorylated SET was detected by Western blotting using anti-SET antibody. Results are expressed as the percentage of SET protein level in absence of treatment. Results are expressed as mean ± SEM from 3 independent experiments. Data were analyzed by Two-way ANOVA followed by Tukey’s test, with distinct letters indicating significant differences between treatments (p<0.05). (c) αT3-1 gonadotrope cells were pre-incubated or not with the PKC inhibitor GF109203X (2 μM, 1 hour) prior to GnRHa stimulation (100 nM, 0.5 hour) and SET protein expression was detected by Western blotting using anti-SET antibody. Results are normalized by vinculin signals and expressed as the percentage of SET protein level in absence of treatment. Results are expressed as mean ± SEM from 3 to 4 independent experiments. Data were analyzed by Two-way ANOVA followed by Tukey’s test, with distinct letters indicating significant differences between treatments (p<0.05).

SET has been described as a phosphoprotein in numerous human cell lines [39]. Here, we demonstrated that SET is also expressed in murine gonadotrope cells as a phosphorylated protein. Indeed, SET immunoprecipitated from [^32^P]-orthophosphate metabolically labelled αT3-1 gonadotrope cells was loaded with radioactive phosphate as evidenced by autoradiography (Fig 2A, *left panel*). This was confirmed by an alternative experimental approach, allowing enrichment of phosphoproteins by chromatography. Indeed, we observed that phosphoprotein purification columns loaded with unstimulated cells lysates retained significant amounts of phosphorylated SET protein (Fig 2A, *right panel*). Interestingly, a short-term stimulation of cells with GnRHa (0.5 hour) significantly increased phosphorylation of SET as evidenced by an increase of the radioactivity contained in the immunoprecipitated SET (Fig 2A, *left panel*, ~7-fold increase) and a significant increase of the amount of SET retained on phosphoprotein purification column (Fig 2A, *right panel*, ~2-fold increase). Enrichment of ERK1/2 protein on phosphoprotein purification columns under GnRHa stimulation, which reflects GnRHa-induced ERK1/2 phosphorylation, was used as a control of activation of GnRHR signaling (Fig 2A, *right panel*). Altogether, data generated by two different approaches demonstrate that GnRH increases SET phosphorylation. Pre-incubation of cells with the PKC inhibitor, GF109203X, significantly decreased basal phosphorylation of SET (Fig 2B, 32 ± 1% of untreated cells), demonstrating that SET phosphorylation is mediated through activation of PKC. Interestingly, GnRHa-induced SET phosphorylation (Fig 2B, 194 ± 11% of untreated cells) was abolished by pre-incubation of cells with the PKC inhibitor, indicating that GnRH-induced SET phosphorylation is mainly mediated through activation of PKC in gonadotrope cells (Fig 2B, 32 ± 1 *vs*. 42 ± 3% of untreated cells, in cells pre-treated with GF109203X, in absence or presence of GnRHa, respectively; non significant). Moreover, GnRHa-induced SET down-regulation (Fig 2C, 60 ± 6% of untreated cells) was abolished by cell pre-incubation with the PKC inhibitor (Fig 2C, 87 ± 7 *vs*. 99 ± 20% of untreated cells, in cells pre-treated with GF109203X, in absence or presence of GnRHa, respectively; non significant). Altogether, these results indicate that GnRHa-induced SET protein phosphorylation and down-regulation are mediated through PKC-dependent mechanisms.

### Involvement of serine 9 in SET targeting into proteasome

**Fig 3:**
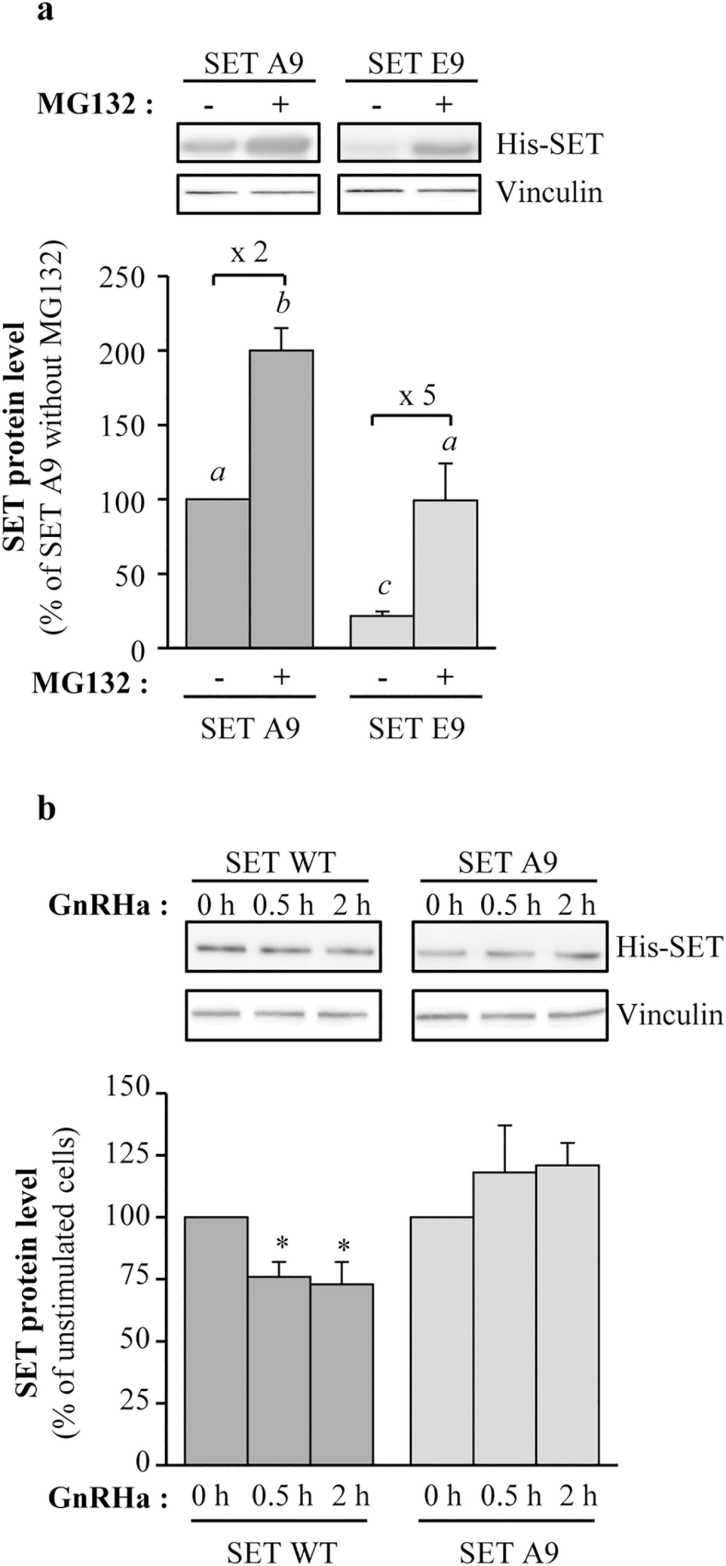
Impact of serine 9 phosphorylation on SET expression level. **(**a) Gonadotrope cells were transfected either with A9 or E9 His-SET mutants as described in “Materials and methods”. Thirty hours after transfection, cells were pre-incubated (+) or not (-) with MG132 (10 μM, 18 hours). The relative expression levels of both mutants were assessed by Western blotting and normalized by vinculin signals. His-SET proteins migrate on SDS-PAGE at a higher molecular weight than endogenous SET allowing their specific quantification. Results are expressed as the percentage of SET A9 protein level in absence of MG132. Results are expressed as mean ± SEM from 4 to 5 independent experiments. Data were analyzed by Two-way ANOVA followed by Tukey’s test, with distinct letters indicating significant differences between treatments (p<0.05). (b) Gonadotrope cells were transfected either with the wild type His-SET or the His-SET A9 mutant and were treated or not with GnRHa (100 nM) for 0.5 or 2 hours. SET expression level was assessed by Western blotting and normalized by vinculin signals. Results are expressed as the percentage of SET WT or SET A9 protein levels in absence of GnRHa. Results are expressed as mean ± SEM from 4 independent experiments. Data were analyzed by One-way ANOVA followed by Dunnett’s test*: p<0.05, compared to no GnRHa.

Among the serines potentially phosphorylated in SET, we focused our study on serine 9, which is phosphorylated in a cellular context and belongs to a PKC consensus phosphorylation site [8,39,40]. In order to determine the impact of serine 9 phosphorylation on SET expression level, we used mutants of a tagged form of SET, His-SET, where serine 9 was mutated to either alanine (A9) to mimic the non-phosphorylatable form or to glutamic acid (E9) to mimic the phosphorylated form [26]. These two mutants have been previously used to highlight the role of serine 9 phosphorylation in diverse aspects of SET regulation [8,26,40,41]. We transfected gonadotrope cells with equal amount of the respective expression vectors and quantified the protein levels of mutant forms SET A9 and E9 by Western blot. Interestingly, mutants displayed very distinct expression profiles as the phospho-mimetic SET E9 mutant was approximately 5 times lower expressed than the non-phosphorylatable form A9 (Fig 3A, 22 ± 3% of the SET A9 mutant expression level). Incubation of gonadotrope cells with the proteasome inhibitor, MG132, markedly increased the expression of the SET mutant E9 restoring its expression to the expression level of the SET A9 mutant (Fig 3A, 99 ± 25% of the SET A9 mutant expression level). MG132 also increased, but to a much lesser extent, SET A9 mutant expression level indicating that other mechanisms may contribute to target SET into the proteasome pathway (200 ± 15% as compared to no MG132). Such differential sensitivity to MG132 between the two mutants strongly suggests that phosphorylation of SET on serine 9 plays a critical role in SET down-regulation through proteasome. If such a hypothesis is relevant, GnRHa should be less effective in reducing the expression of the non-phosphorylatable SET A9 form. We thus compared the effect of GnRHa on wild type and A9 forms of SET. GnRHa stimulation decreased expression of wild type SET with a similar kinetics and extent as for endogenous SET (Fig 3B, 76 ± 5% and 73 ± 8% compared to unstimulated cells, at 0.5 and 2 hours, respectively). Interestingly, the non-phosphorylatable A9 form was resistant to GnRHa-induced down-regulation (Fig 3B, 117 ± 27% and 128 ± 12% compared to untreated cells, at 0.5 and 2 hours, respectively). Altogether, these results suggest strongly that SET phosphorylation on serine 9 is crucial for GnRH-induced degradation of SET through proteasome.

### SET is down-regulated under physiological GnRH stimulation–Evidence in prepubertal female mice pituitaries

**Fig 4:**
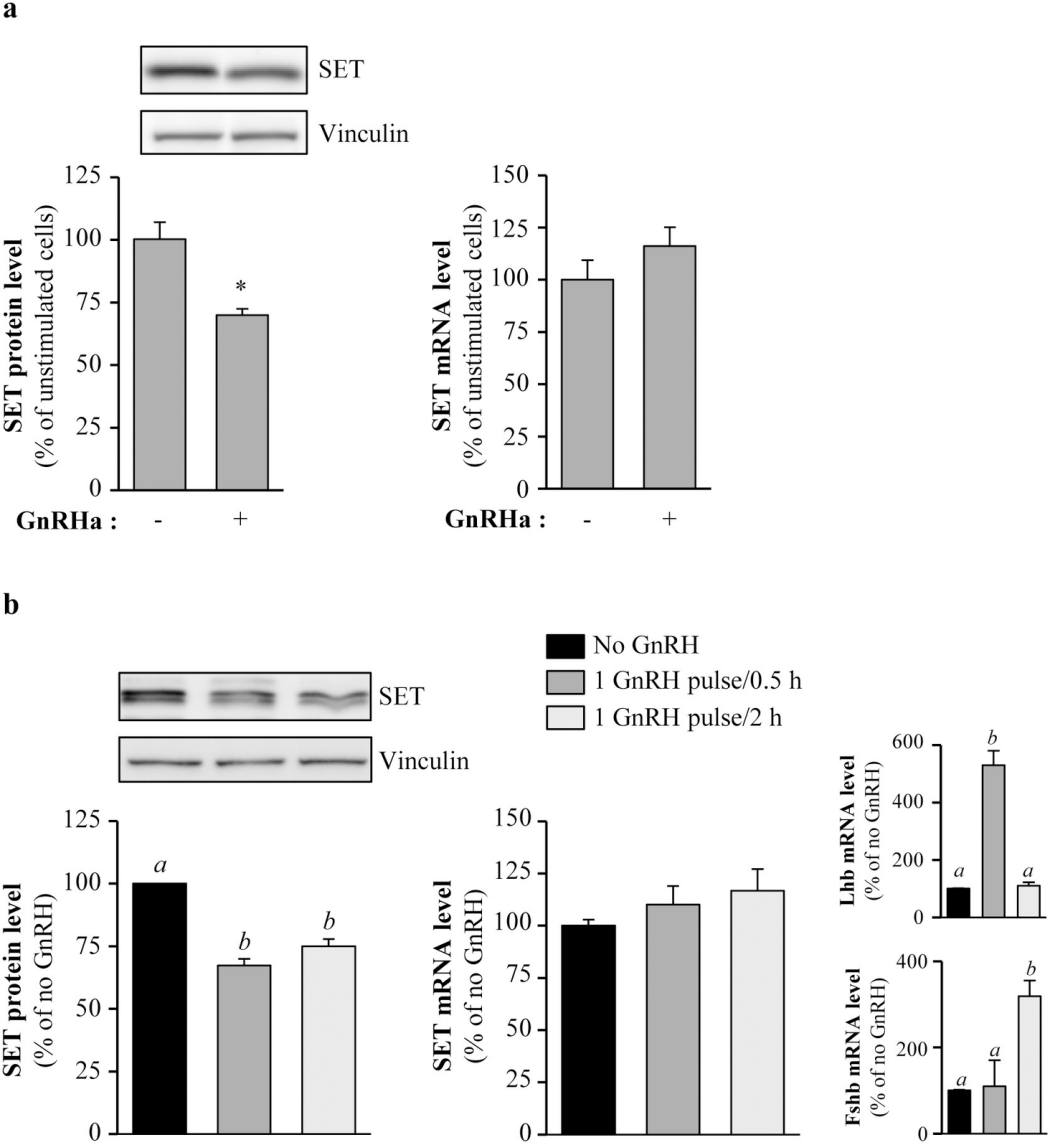
Pulsatile native GnRH induces SET protein down-regulation in LβT2 cells. (a) LβT2 gonadotrope cells were treated with GnRHa (100 nM) for 2 hours and SET protein (*left panel*) and SET mRNA (*right panel*) levels were determined by Western blotting and by real-time RT-PCR, respectively. A representative immunoblot of SET expression is shown. Results are normalized by vinculin signals (SET protein) or by mRNA levels of *Hprt* (SET mRNA) and are expressed as percentage of the amount of SET in unstimulated cells. Results are expressed as mean ± SEM from 3 independent experiments. Data were analyzed by t test for unpaired groups *: p<0.05, compared to no GnRHa. (b) LβT2 gonadotrope cells were cultured in perifusion chambers as described in “Materials and methods” and challenged or not with pulsatile GnRH (10 nM) at high and low frequencies (one pulse every 0.5 hour or one pulse every 2 hours, respectively). At the end of the incubation, proteins and mRNA were extracted and SET protein and *Set*, *Lhb and Fshb* transcripts levels were determined by Western blotting and by real-time RT-PCR, respectively. A representative immunoblot of SET expression is shown. Results are normalized by vinculin signals (SET protein) or by mRNA levels of *Hprt* (*Set*, *Lhb and Fshb* mRNA) and expressed as percentage of the amount of SET in unstimulated cells. Results are expressed as mean ± SEM from 3 independent experiments. One-way ANOVA followed by Tukey’s test, ***: p<0.001, compared to no GnRH.

To address the physiological relevance of GnRH regulation of SET protein expression, we first asked if such regulation was also observed in the LβT2 gonadotrope cell line, which is more differentiated than the αT3-1 cell line. As observed in αT3-1 cells, GnRHa treatment (100 nM, 2 hours) decreased SET protein expression levels in LβT2 cells by ~30% (Fig 4A, *left panel*, 70 ± 2% of unstimulated cells) without altering SET mRNA level (Fig 4A, *right panel*). To mimic GnRH stimulations *in vivo*, we stimulated LβT2 cells plated in perifusion chambers with the native neurohormone GnRH (10 nM) in a pulsatile way, at high (1 pulse/0.5 hour) or low (1 pulse/2 hours) frequencies. As expected mRNA levels of the specific beta subunits of LH (*Lhb*) and FSH (*Fshb*) were specifically increased upon high- and low-frequencies stimulations, respectively (High-frequency: *Lhb*: 5.3 ± 0.5 fold over unstimulated cells and *Fshb*: 1.1 ± 0.6 fold over unstimulated cells; Low-frequency: *Fshb*: 3.19 ± 0.36 and *Lhb*: 1.1 ± 0.12 fold over unstimulated cells, Fig 4B, *right panel*). Interestingly, native GnRH delivered in a pulsatile way induced a ~25–30% decrease in SET protein expression at both high- and low-frequencies (Fig 4B, 67 ± 3% and 75 ± 3% of unstimulated cells, at high- and low-frequencies, respectively). This occurred without any significant change in SET mRNA levels (Fig 4B). These results suggest that SET protein down-regulation could occur *in vivo* under pulsatile GnRH stimulations.

**Fig 5:**
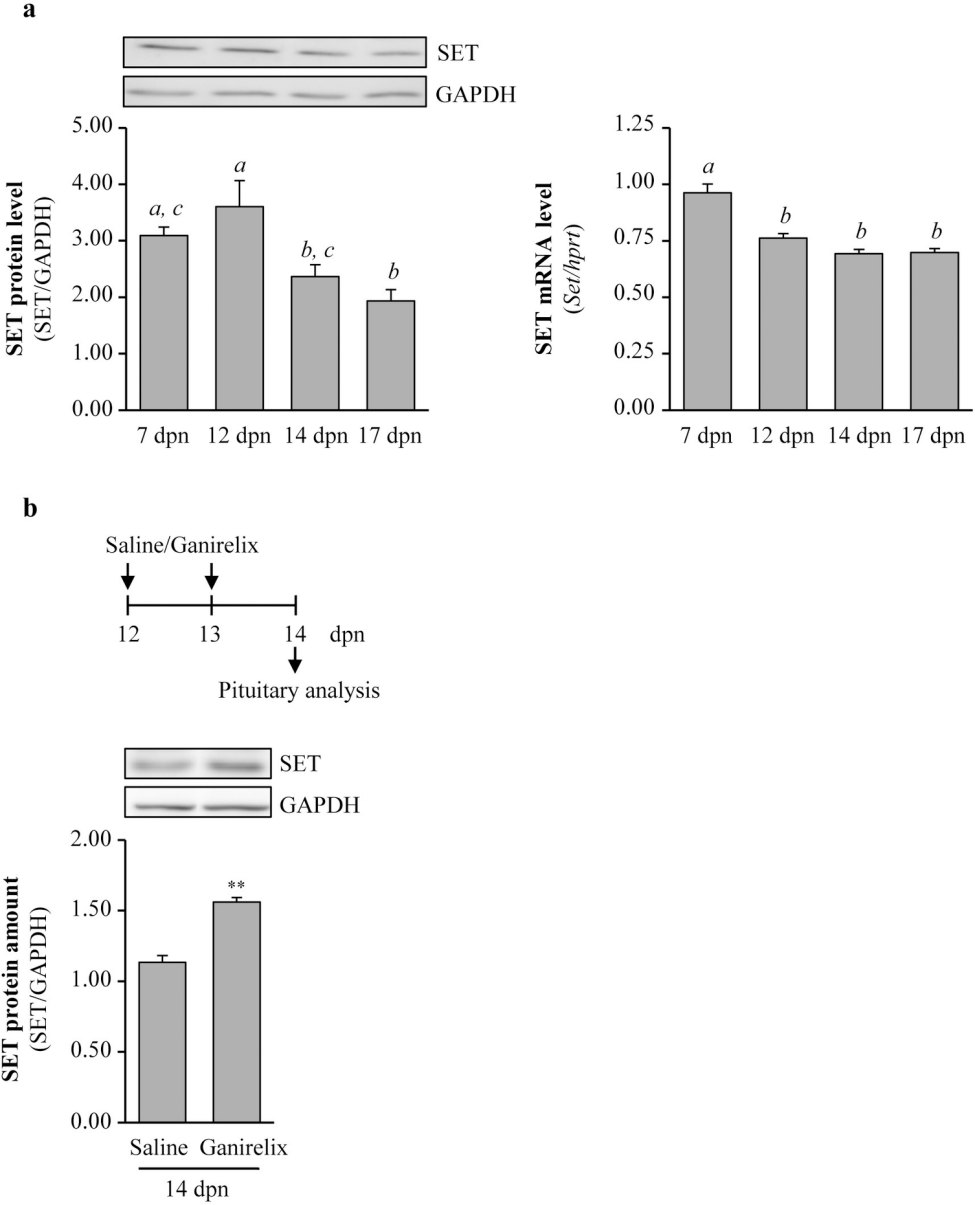
GnRH induces SET protein down-regulation in infantile mice pituitary. (a) *Left panel*—SET protein abundance was analyzed in infantile pituitaries (7–17 dpn) by Western blotting, with GAPDH used as a loading control. Bar graphs show the mean ± SEM of SET levels normalized to those of GAPDH (n = 3 to 8 pituitaries/age). A representative immunoblot of SET expression is shown. Data were analyzed by one-way ANOVA, followed by Tukey’s test with distinct letters indicating significant differences between ages (p<0.05). *Right panel*—The relative pituitary abundance of SET mRNA in infantile (7–17 dpn) females was determined by real-time RT-PCR and normalized to the mRNA levels of *Hprt* (n = 4 to 8 pituitaries/age). Bar graphs show the mean ± SEM of relative quantification. Data were analyzed by one-way ANOVA, followed by Tukey’s test with distinct letters indicating significant differences between ages (p<0.05). (b) SET protein abundance was analyzed in infantile pituitaries after treatment with the GnRH antagonist Ganirelix or saline vehicle at 12 and 13 dpn. A representative blot is shown. Bar graphs show the mean ± SEM of SET levels normalized to those of GAPDH (n = 3 to 5 pituitaries/condition). Data were analyzed by t test for unpaired groups, **: p<0.01, compared to 14 dpn saline.

To address such regulation *in vivo*, we used the infantile mouse as a model to study GnRH action. Indeed, during the early postnatal developmental stage in mice (first 3 weeks of life) there is an increase in GnRH neurons activity [42] associated with high circulating levels of both FSH and LH that we previously observed around 12–14 days postnatal (dpn) [37]. We observed that SET protein could be easily detected in the pituitary throughout the infantile period (Fig 5A, *left panel*). Interestingly, there was a significant down-regulation of SET relative abundance between 12 and 14 dpn (34 ± 6% decrease), at the time when both LH and FSH are highly synthesized [37]. SET mRNA levels significantly decreased between 7 dpn and 12 dpn and remained stable from 12 dpn to 17 dpn (Fig 5A, *right panel*). Therefore, the decrease in SET protein levels between 12 and 14 dpn unlikely results from a down-regulation in mRNA levels since they remained unchanged at this time (Fig 5A, *right panel*). To clarify the role of GnRH in the decline of SET protein abundance *in vivo*, we treated infantile mice at 12 dpn and 13 dpn with a GnRH antagonist, *i*.*e*. the Ganirelix, at a dose that significantly blocks GnRH action as we have shown by the dramatic reduction in both FSH and LH circulating levels [37]. Importantly, there was a significant elevation of SET relative protein levels in Ganirelix-treated mice at 14 dpn as compared with their saline controls (Fig 5B, 38 ± 5% increase). Hence, these data suggest that the regulation of SET protein levels *in vivo* is driven, at least in part, by GnRH.

## Discussion

Despite the major role played by SET in a large variety of cellular and physiological functions, the factors and mechanisms involved in the regulation of its expression are currently poorly known. The very few studies describing factors modulating SET expression are restricted to the effects of exogenous molecules such as chemotherapy drugs (paclitaxel, paeonol and the mushroom Ganoderma lucidum [43,44,45]) and a chemical pollutant (trichloroethylene [46]). Here, we report the identification of the first SET endogenous regulating signal, *i*.*e*. the neurohormone GnRH. This was demonstrated in two gonadotrope cell lines as well as *in vivo* in the pituitary of infantile mice.

Importantly, we showed that GnRH rapidly and sustainably decreases SET protein expression level in gonadotrope cells *in vitro* without altering mRNA abundance. This was observed in response to the GnRH agonist, triptorelin, as well as in response to native GnRH administered in a pulsatile way to mimic *in vivo* stimulations of pituitary gonadotrope cells. GnRH-induced decrease in SET expression similarly occurred at high- and low-frequencies of GnRH stimulations indicating that GnRH regulation of SET expression is insensitive to GnRH pulse frequency. This contrasts with the expression of gonadotropins LH and FSH occurring preferentially and respectively at high- and low-frequencies. This suggests that SET level expression does not probably contribute to the decoding of GnRH pulsatility by gonadotrope cells. We also report that SET expression decreases shortly after birth in the female pituitary, concomitantly to the activation of GnRH neurons, and that SET protein expression is driven, at least in part, by GnRH. Using cell-based and biochemical approaches, we have deciphered a post-translational mechanism driving its down-regulation that involves a rapid degradation of SET protein through the proteasome pathway. SET targeting to the proteasome is consistent with the fact that we have identified, using ubpred (http://www.ubpred.org), five lysines in the mouse SET protein sequence (K11, K26, K141, K154 and K180) that are susceptible to be ubiquitylated. Moreover, in different proteomic studies, SET has been shown to interact with proteins of the ubiquitylation machinery such as MIB2 [47], MDM2 [48], PARK2 [49], Huwe1 [50]and UBR5 [51]. SET is not the only protein in gonadotrope cells to be targeted by GnRH for degradation to the proteasome pathway. Indeed, this has been also described for inositol 1,4,5-trisphosphate receptors [52] and several PKC isoforms [53,54] and this contributes to desensitization of GnRHR-dependent calcium signaling.

We also demonstrated that GnRH increases SET phosphorylation. To date, there is almost no insight into the nature of factors and mechanisms involved in SET phosphorylation. To our knowledge, only two other GPCRs, the β1-adrenergic receptor and type 1A angiotensin receptor, have been shown to induce SET phosphorylation [8,9]. In our study, we demonstrated that GnRH induces SET phosphorylation through PKC. Phosphorylation of SET, in particular on serine 9, is involved in the regulation of SET subcellular localization as well as in SET interaction with other proteins such as importins, Rac1 and PP2A [8,26,40]. Serine 9 phosphorylation induces translocation of SET from the nucleus to the cytoplasm [41] and is responsible for the abnormal accumulation of SET in the cytoplasm of neurons from patients with Alzheimer’s disease [40]. Interestingly, casein kinase 2 has been proposed to phosphorylate SET at serine 9 in Alzheimer disease resulting in tau hyperphosphorylation and cognitive impairements [55]. Here, we demonstrate that serine 9 phosphorylation also mediates GnRH-induced SET degradation through proteasome. Indeed, a serine 9 phosphorylation-defective SET mutant is resistant to degradation following a short treatment with GnRH. This suggests strongly that GnRH-induced SET phosphorylation on serine 9 initiates SET targeting to proteasome. Despite this critical role of serine 9, we cannot exclude the possibility that SET might be phosphorylated on additional sites upon GnRH stimulation. Three other serines in SET, in position 24, 93 and 171, can be phosphorylated in a cellular context [8,39,56]. Unfortunately, we and others were unable to identify by mass spectrometry the nature of the phosphorylated serines in SET [8]. This is probably due to technical limitations to detect under-represented phosphorylated peptides or to the loss of some SET peptides during analytical process, notably the amino-terminal SET peptide containing serine 9, which was never detected in our mass spectrometry studies.

A deregulation of SET expression has been clearly associated with the development of pathologies such as cancers and neurodegenerative diseases. Indeed, overexpression of SET has been found in numerous human cancers [32,33,34,57] and in brains of patients with Alzheimer’s disease [35]. Here, we showed that SET protein down-regulation is driven by the major regulator of reproductive function, *i*.*e* the neurohormone GnRH. Of interest, GnRHR is expressed in many tumor tissues [58] in which SET has been involved in the tumor progression and aggressiveness. Moreover, GnRH has been shown to inhibit cell proliferation in many cell types and to have a potent anti-tumor effect in tumors from ovary, prostate, breast, pancreas, colon, kidney [58]. Given our demonstration that GnRH decreases SET protein expression in pituitary gonadotrope cells, one could hypothesize that GnRH anti-tumor effect could be driven at least in part by an inhibition of SET expression. This remains to be explored and our study might have unveiled a potential new mechanism involved in the anti-tumor effect of GnRH. GnRHR is also expressed in several regions of the brain [59,60]. Interestingly, increase of SET expression in brains of patients with Alzheimer’s disease has been associated with the accumulation of phosphorylated Tau in neurons through inhibition of PP2A activity by SET [61,62,63]. It might be of interest to determine whether GnRH would decrease SET expression in neurons, as observed here in gonadotrope cells, hence contributing to reduce accumulation of phosphorylated Tau.

Furthermore, our study suggests that GnRH-induced down-regulation of its receptor accessory protein SET may contribute to the regulation of pituitary gonadotrope activity notably through the fine-tuning of GnRHR-dependent cAMP signaling. We previously down-regulated endogenous SET protein expression, using small interfering RNA strategy, to a similar extent as the one observed in response to GnRH. We have shown that such SET down-regulation decreases GnRHR coupling to cAMP pathway in gonadotrope cells and leads to abrogation of GnRH-induced *Gnrhr* gene transcription, which depends on cAMP mobilization by GnRH [3]. These findings suggest that GnRH-induced SET down-regulation represents a regulatory loop between GnRHR and its accessory protein SET, thereby providing a novel mechanism for GnRHR desensitization. This currently needs to be explored and should be addressed in future studies. The original finding that SET expression decreases in female mice pituitary during the infantile period might not only impact GnRHR signaling in pituitary gonadotropes but may also alter the ability of SET to regulate gene transcription as well as many cellular processes through inhibition of PP2A. Although this remains to be explored, SET regulation of many cellular functions suggests that its expression decrease during the infantile period might impact pituitary function.

In conclusion, our study identifies the first hormonal regulator of SET expression and provides new insights into the mechanisms driving SET regulation. This may help to understand how SET is physiologically regulated but also how it is deregulated in cancer and Alzheimer’s disease.
